# The pattern of collagen production may contribute to the gluteal muscle contracture pathogenic process

**DOI:** 10.1186/s13018-023-04069-w

**Published:** 2023-08-08

**Authors:** Xiaocheng Jiang, Hang Zhang, Yuxiang Ren, Li Yang, Ling Zhong, Jiang Guo, Xintao Zhang

**Affiliations:** Peking University Shenzhen Hospital, Shenzhen City, China

**Keywords:** Gluteal muscle contracture, Arthroscopy, Relapse, Collagen, Muscle repair

## Abstract

**Introduction:**

Arthroscopic release is now the gold standard globally for gluteal muscle contracture (GMC) treatment. However, some patients fail to improve after the first operation and are forced to undergo a second operation. This study explores the essential role collagen fibers may play in muscle contracture in GMC.

**Methods:**

From February 2010 to May 2018, 1041 hips of 543 GMC patients underwent arthroscopic release. Among them, 498 (91.7%) patients had bilateral GMC and were admitted to the retrospective cohort study. Pathological testing and type III collagen testing were used in contracture tissue studies. Single-cell RNA-sequencing analysis was applied to explore the role of fibroblasts in muscle repair.

**Results:**

Compared with GMC II patients, GMC III patients displayed higher clinical symptoms (*P* < 0.05). Six weeks after the surgery, the patients in GMC II had a lower prominent hip snap rate, higher JOA score, and better hip range of motion (*P* < 0.05). Compared with normal muscle tissue, contracture-affected tissue tended to have more type III collagen and form shorter fibers. Recurrent GMC patients seemed to have a higher type III collagen ratio (*P* < 0.05). In contrast to normally repairable muscle defects, fibroblasts in non-repairable defects were shown to downregulate collagen-related pathways at the early and late stages of tissue repair.

**Discussion:**

This study describes the arthroscopic release of GMC. Study findings include the suggestion that the collagen secretion function of fibroblasts and collagen pattern might influence the muscle repair ability and be further involved in the GMC pathogenic process.

**Supplementary Information:**

The online version contains supplementary material available at 10.1186/s13018-023-04069-w.

## Introduction

Gluteal muscle contracture (GMC) is a clinical syndrome caused by fibrosis and necrosis of the gluteal muscles and fasciae [1]. Patients typically present with abduction and external rotation of the hip and cannot bring the knees together while squatting [2]. Usually, the onset is bilateral (in fact, unilateral GMC is relatively rare), and men have a higher incidence than women. In China, in particular, the incidence rate of GMC ranges from 1 to 2.5% [3]. Two treatment options for GMC are non-operative or operative management. Non-operative management includes massage, physiotherapy, shortwave diathermy, and active and passive stretching exercises [4]. In contrast, operative management includes open surgery [5], needle knife [6], and arthroscopic release [7] from the time of development. The latter was introduced as a minimally invasive technique and has dramatically gained popularity among orthopedic surgeons [7, 8]. Several reports have highlighted the good practice of arthroscopic release and its advantages over open surgery [2, 9, 10]. In our practice, compared with open surgery, arthroscopic surgery offers the advantages of smaller wounds, faster recovery, less bleeding, and faster operation time. A skilled surgeon who applies arthroscopic surgery can see a deeper contracture band, and the release will be more thorough, which is very important in severe cases. The open surgery was previously the first-class treatment choice, arthroscopic release is now the global gold standard [8]. Based on the experience of surgical release in Uganda, children with GMC can continue to benefit at 5-year follow-up [11]. In clinic, we observed that patients who were more severe at the onset of the disease seemed to have a more severe prognosis. We retrospectively summarized the clinical cases of GMC with different severity to intuitively display the changes of symptoms of GMC patients after arthroscopic release.

During the development of arthroscopic gluteal muscle release in the past years, we have noticed that many patients failed to achieve satisfactory improvement after the first operation and were forced to undergo a second operation—such patients in clinical settings account for about 5–10% of the total cases. They needed second surgery within 0.5–2 years after their first arthroscopic surgery. These less-than-optimal outcomes beg the question of what factors cause relapse and how the contracture tissue changes after relapse. Therefore, we engaged in detailed pathological studies to investigate factors leading to secondary surgery in patients with grade II/III GMC [7, 12] while aiming to identify the key factors associated with recurrence.

In addition to its high recurrence rate, understanding of the complex pathology and etiology of GMC remains limited. Some previous reports suggested that repeated intramuscular injections at a younger age might be the most likely culprit in gluteal muscle contracture [8, 13]. However, according to a recent systematic analysis that examined past scholarly investigations of injections as a possible distant cause of GMC, 90% of studies cited previous case reports, and only two studies examined whether injections and GMC were causally related [1]. Other mechanisms, such as inflammation and immunology, may also play roles in developing this disease. One study reported that collagen types I and III, along with TGF-β1 and TGF-β3, were up-regulated in GMC patients’ biopsy specimens, demonstrating the potential of collagen involvement in the disease process [14]. In line with this finding, we hoped to find possible etiologies by conducting pathological analysis of biopsies from patients with recurrent GMC. Prior studies of skeletal muscle fibrosis have suggested that faulty repair patterns after muscle tissue damage may cause muscle fibrosis and contractures. Along those lines, the excessive accumulation of ECM components, especially collagen, participates in skeletal muscle fibrosis and exacerbates muscle damage [15, 16]. Injections result in tiny wound defects, which initiate tissue repair. We hypothesized that the injection, especially the inappropriate solvent (benzyl alcohol), will interfere with the normal recovery of muscles and cause irreversible damage. Since muscle fibrosis may be associated with failed tissue repair processes, the relationship between muscle fibrosis in GMC patients and the tissue repair process is worth exploring.

## Materials and methods

### Case collection

From February 2010 to May 2018, 1041 hips of 543 GMC patients underwent arthroscopic release at the Department of Sports Medicine and Rehabilitation, Peking University Shenzhen Hospital. Among them, 498 (91.7%) patients with bilateral GMC were admitted to the subsequent analysis. The diagnostic criteria for these GMC patients were based on the history of repeated intramuscular injections, having obvious GMC symptoms, including girdle-like contracture of the iliotibial band, knee pain, weak knees upon standing, and varying degrees of shifting of the patella outward when squatting. Magnetic resonance imaging (MRI), X-ray, and necessary physical examination are essential reference evidence in clinical diagnosis. Inclusion criteria covered the following: (1) bilateral buttocks with Grade II (The extorsion of lower limb is moderate, the abduction contracture ranges from 15° to 60° with both hip and knee joint in 90° of flexion or adduction range is less than 10° with no flexion. Ober's sign and frog squatting sign are positive. The limp gait is apparent with lateral inclination of pelvis on anteroposterior radiograph being less than 20°) or bilateral buttocks with Grade III (The extorsion of lower limb is severe, the abduction contracture is more than 60° with both hip and knee joint in 90° of flexion or adduction range is less than 0° with no flexion. Ober’s sign and frog squatting sign are strongly positive. The limp gait is remarkably apparent with lateral inclination of pelvis on anteroposterior radiograph being more than 20°) [12], (2) operated on under intravertebral anesthesia by the same surgeon group, and (3) no history of serious underlying diseases. Exclusion criteria were as follows: (1) radiographic evidence of hip dislocation or hip dysplasia by X-ray examination, (2) clinical manifestations of neurological damage, (3) diagnosed with gluteal soft tissue tumors, (4) gluteal compartment syndrome, and (5) follow-up period shorter than 24 months. The ethics committee approved the studies of the Peking University Shenzhen Hospital. Written informed consent for medical record review was obtained from all patients following the Declaration of Helsinki.

### Arthroscopy release and rehabilitation procedure

**Fig. 1:**
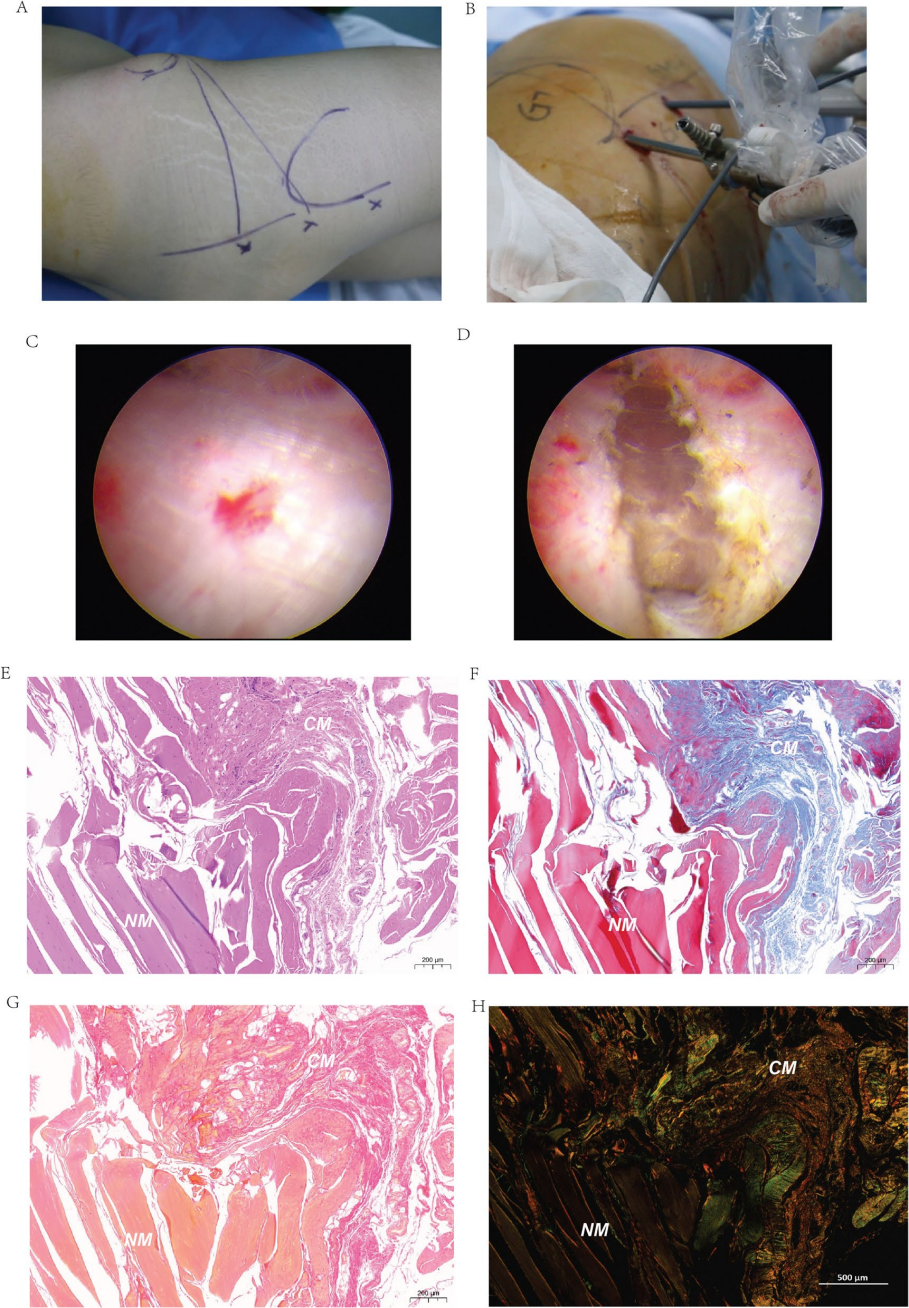
Histopathological examination of contracture sites in patients with GMC. Three-step arthroscopic gluteal muscle release method: **A** The triangle area is the artificial cavity area, the left *x* is the arthroscopic observation approach, the middle *x* is the arthroscopic operation approach, and the right *x* is the arthroscopic-assisted operation approach (this approach may be required for very severe gluteal muscle patients); **B** Arthroscopic operation; **C** Contracture zone in the arthroscopic field of vision; **D** After the release of the contracture band in 1c; E. HE staining result; F. Masson staining result; **G** Sirius red staining result; **H** Sirius red staining result under polarized light. CM is contracture muscle; NM is normal muscle. The scale for E–G is 200 um. The scale for H is 500 um

The patient was placed in the lateral position following intravertebral anesthesia. One entrance was located on the top surface of the greater trochanter, and the other entrance was 4 cm at the distal end of the greater trochanter. A blunt cut was selected between the subcutaneous fascia and GMC tissue to form a subcutaneous triangular space from these two entrances to the anterior superior iliac spine (ASIS) (Fig. 1A, B). After flushing the space with saline, a shaver and radiofrequency were used to remove fat and fibrous tissue. Next, a three-step method was used to release the superficial and deeper contracture tissue. The contracture tissue that appeared silver and filamentous under arthroscope was removed and gasified by radiofrequency ablation (Fig. 1C). The first step involved using radiofrequency to release the tensor fascia’s thickened fascia and scar contracture zone. The range of issuance started from ASIS and continued to the greater trochanteric. The second step began from the front edge of the gluteus maximus and extended to the curved line along the distal femur. Lastly, in the third step, the gluteus medius and gluteus minimus were released (Fig. 1D). In particularly severe cases of GMC, the external rotators and hip joint capsule were also released. Usually, intramuscular probing and cutting were also required. After the release was complete, a check of the passive flexion, internal and external rotation, abduction, and adduction of the hip was performed to ensure that the hip joint had returned to the normal range of motion and observe whether the bounce sound had disappeared. Hemostasis measures were used throughout the operation to provide a clear field of vision and help avoid postoperative hematoma. Rehabilitation started 24 h after surgery. Functional exercises, including (1) crouching with both knees close to each other, (2) abducting and adducting the upper limbs while lying in a lateral position, and (3) lying on the back, bringing the knees to the chest, clasping the hands to the front of the shin, and internally rotating the hips while keeping the pelvis as flat as possible, were performed to prevent scar formation and muscle wasting. The patients performed these exercises three to five times daily with 20–30 repetitions depending on their endurance. The average length of hospital stay was 7.1 ± 0.8 days (range, 4–15 days) after surgery. All patients were followed at regular intervals after surgery. For the first 2 months, they were reassessed every 2 weeks to monitor wound complications and examine the hips’ function. After that initial period, they were seen for follow-up every 3 months.

### Clinical index measurement

We retrospectively reviewed the medical database to extract possible key factors before the first operation (preoperative factors) or 6 weeks after the first operation (postoperative factors). Preoperative factors included onset age, gender, GMC class (II/III), and the history of repeated intramuscular injections (Yes/No), JOA score for hip joint (bilateral mean). Postoperative factors included JOA score for hip joint (bilateral mean). The JOA hip scaling system consists of four subcategories: pain (Pain), range of motion (ROM), ability to walk (Gait), and activities of daily life (ADL) [17]; obvious hip snap (Yes/No) [10, 11]; subcutaneous induration (Yes/No); hip range of motion (0:0–120; 1:121–150; 151–200; 201–220) (bilateral mean) [18]. The calculation of the single hip range of motion was equal to the sum of the mobility of flexion (140°), adduction (40°), and internal rotation (40°).

### Histopathological examination

Segments from the contracture site were fixed in PFA (4%), embedded in paraffin and then cut into 3-μm-thick sections. Hematoxylin and eosin (HE) staining was used for pathological purposes (Fig. 1E). Masson staining and Sirius red staining were used for staining and differentiation of collagen fibers (Fig. 1F, G). We used an optical microscope (Eclipse CI-l, Nikon, Japan) to take photos of the slices and a pathological section scanner (panoramic desk/midi/scan/250, Japan) to scan the whole pathological section. For the Sirius red-stained sections, we used a polarized light photo-graphing microscope (Eclipse CI-l, Nikon, Japan) to select the target area of the tissue for 200 X imaging (Fig. 1H). When imaging, we tried to fill the whole field of vision with the tissue to ensure that the background light for each photo was consistent. After imaging, we used the Image-Pro Plus 6.0 analysis software to set the pixel area as the standard unit uniformly and measure the pixel area of type I collagen in three fields of view in each slice as well as the corresponding type III collagen pixel area. In addition, we calculated the pixel area ratio of type I and type III collagen = type I collagen pixel area/type III collagen pixel area.

### Human type III collagen concentration measurement

The contracture tissue taken during arthroscopic surgery was washed three times with normal saline. Next, the contracture tissue was separated into small 1 g pieces and put into liquid nitrogen for quick freezing. Before testing, the tissue was taken out, and after grinding, 2 ml 1X PBS was added for the preparation of the tissue suspension. The Human Col III ELISA Kit (CSB-E04799h, CUSABIO, China) was used in this study. The standard curve was calculated according to the manufacturer’s instructions.

### Bioinformatic analysis for single-cell RNA sequencing data

The public database GSE163376 was used to study the changes in fibroblasts during wound healing [19]. R package Seurat v3.1.2 was employed for dimension-reduction and clustering [20]. All gene expression was normalized and scaled using the functions “Normalize Data” and “Scale Data.” The top 2000 variable genes were selected by “Find Variable Features” for PCA analysis. Cells were separated by “Find Clusters” using the top 20 principal components and a resolution parameter of 2.0 [21]. For the sub-clustering of 12 cell types, we set the resolution at 1.2. The UMAP algorithm was applied to visualize cells in a two-dimensional space. Differentially expressed genes (DEGs) analysis was performed using Seurat v3.1.2. Cluster marker genes were determined with Seurat (only.pos = T, min.pct = 0.25, logfc.threshold = 0.25, return.thresh = 0.01) and scCATCH (species = ‘Mouse’, match_CellMatch = T, tissue = ‘Muscle’, cell_min_pct = 0.25, logic = 0.25, *p* value = 0.05) to annotate cell types. Pathway enrichment (including Gene Ontology (GO) and Kyoto Encyclopedia of Genes and Genomes (KEGG)) was done by ‘cluster Profiler’ 4.0 [22].

### Statistical analysis

Quantitative data were presented as means with standard deviations. Categorical data were presented as percentages of the population in groups. The independent sample *t* test or Mann–Whitney *U* test was used for the continuous variables in comparing groups. The chi-square test was used for categorical variables and was corrected when needed. The statistical analyses were performed using the statistical software R × 64 3.6.3, and values of *P* < 0.05 were considered statistically significant.

## Results

### Clinical character summary of the GMC II and GMC III cases with arthroscopic release

The study population included a total of 498 GMC patients who underwent arthroscopic release. To better understand patients’ status concerning different levels of GMC severity, we generated statistics on GMC II and III patients separately. The mean onset age of GMC II was 22.5 ± 10.8 years, and the mean onset age of GMC III was 22.6 ± 10.3 years (*P* > 0.05). There were slightly more women than men in the GMC II and GMC III groups (*P* > 0.05). There was no significant difference between the two groups of patients in terms of whether they had a history of hip injection. In terms of clinical symptoms at the diagnosis checkpoint, GMC III seemed to have a higher ratio of unequal length of lower limbs (80.3%), lower JOA score for hip joint function (74.8 ± 7.2), and worse hip range of motion (0–120°:15.1%) (*P* < 0.05). There was no statistically significant difference in the average time of unilateral surgery between the two groups of patients. The mean time of one-side surgery for GMC II was 26.1 ± 7.9 min, and the mean time of one-side surgery for GMC III patients was or 27.8 ± 11.5 min. At the same time, the postoperative hospital stay of GMC III patients was slightly longer (7.5 ± 0.8, *P* < 0.05). Regarding postoperative recovery, the physical examination of different GMC severity groups revealed significant differences. Six weeks after the surgery, the GMC II patients had a lower obvious hip snap rate, higher JOA score, and better hip range of motion (*P* < 0.05; Table 1).

**Table 1:** Comparison between GMC II and GMC III group

Parameters	GMC II (*n* = 346)	GMC III (*n* = 152)	*P* value
Age (years ± SD)	22.5 ± 10.8	22.6 ± 10.3	0.910*
Gender			0.430
Male (n%)	137 (39.6%)	66 (43.4%)	
Female (n%)	209 (60.4%)	86 (56.6%)	
History of repeated intramuscular injections			0.580
Yes (*n*, %)	330 (95.4%)	147 (96.7%)	
No (*n*, %)	16 (4.6%)	5 (32.9%)	
Unequal length of lower limbs			**0.012**
Yes (*n*, %)	240 (69.4%)	122 (80.3%)	
No (*n*, %)	106 (30.6%)	30 (19.7%)	
Mean time of one-side surgery (min ± SD)	26.1 ± 7.9	27.8 ± 11.5	0.445^#^
Postoperative hospital stay (day ± SD)	6.5 ± 1.2	7.5 ± 0.8	0.019*
Obvious hip snap before first surgery			0.059
Yes (*n*, %)	276 (79.8%)	132 (86.8%)	
No (*n*, %)	70 (20.2%)	20 (13.2%)	
Obvious hip snap after first surgery			**0.004**
Yes (*n*, %)	11 (3.2%)	15 (9.9%)	
No (*n*, %)	335 (96.8%)	137 (90.1%)	
JOA score for hip joint function before first surgery (score ± SD)	79.6 ± 6.4	74.8 ± 7.2	**0.001**^**#**^
JOA score for hip joint function after first surgery (score ± SD)	94.0 ± 3.2	92.5 ± 4.4	** < 0.001**^**#**^
Hip range of motion before first surgery			** < 0.001^**
0–120°	2 (0.6%)	23 (15.1%)	
121–150°	19 (5.5%)	78 (51.3%)	
151–200°	290 (83.8%)	49 (32.2%)	
201–220°	35 (10.1%)	2 (1.3%)	
Hip range of motion after first surgery			** < 0.001^**
121–150°	9(2.6%)	18(5.2%)	
151–200°	22(6.4%)	10(6.6%)	
201–220°	315(91.0%)	124(81.6%)	

*P* value: * Mann–Whitney *U* test, # student *t* test, ^ adjusted chi−square test.

^a^Significant level is 0.05, and significant *P* values are shown in bold.

^b^For the mean time of one-side surgery, JOA score for hip joint function and hip range of motion; if the patient was bilateral, we took the bilateral mean value.

^c^The continuous variables used the independent sample *t* test or Mann–Whitney *U* test, and the classification variables used the chi−square test

### Abnormal collagen pattern in contracture tissue

To tease out the pathological characteristics of the contracture site in patients with GMC, we selected 8 tissue blocks from the patients with GMC III for the first time and 8 tissue blocks from the patients with recurrent GMC III for the experiment. Ten tissue blocks were stained with HE, Masson, and Sirius red. Figure 1A & B illustrates the surgical procedure. The contracture tissue that appeared silver and filamentous under the arthroscope was removed and gasified by radiofrequency ablation (Fig. 1C). After stripping, the contracture was released (Fig. 1D). Compared with normal muscle (NM), the contracture “muscle” (CM) lacked a normal muscle fiber structure and presented part of connective tissue morphology. In addition, the CM area seemed to have more short fibers than long fibers (Fig. 1E). Masson results for the same site revealed that the CM had more collagen fibers than muscle fibers. Sirius red staining showed both type I and type II collagen in the CM area. In contrast, NM seemed to lack type III collagen.

**Fig. 2:**
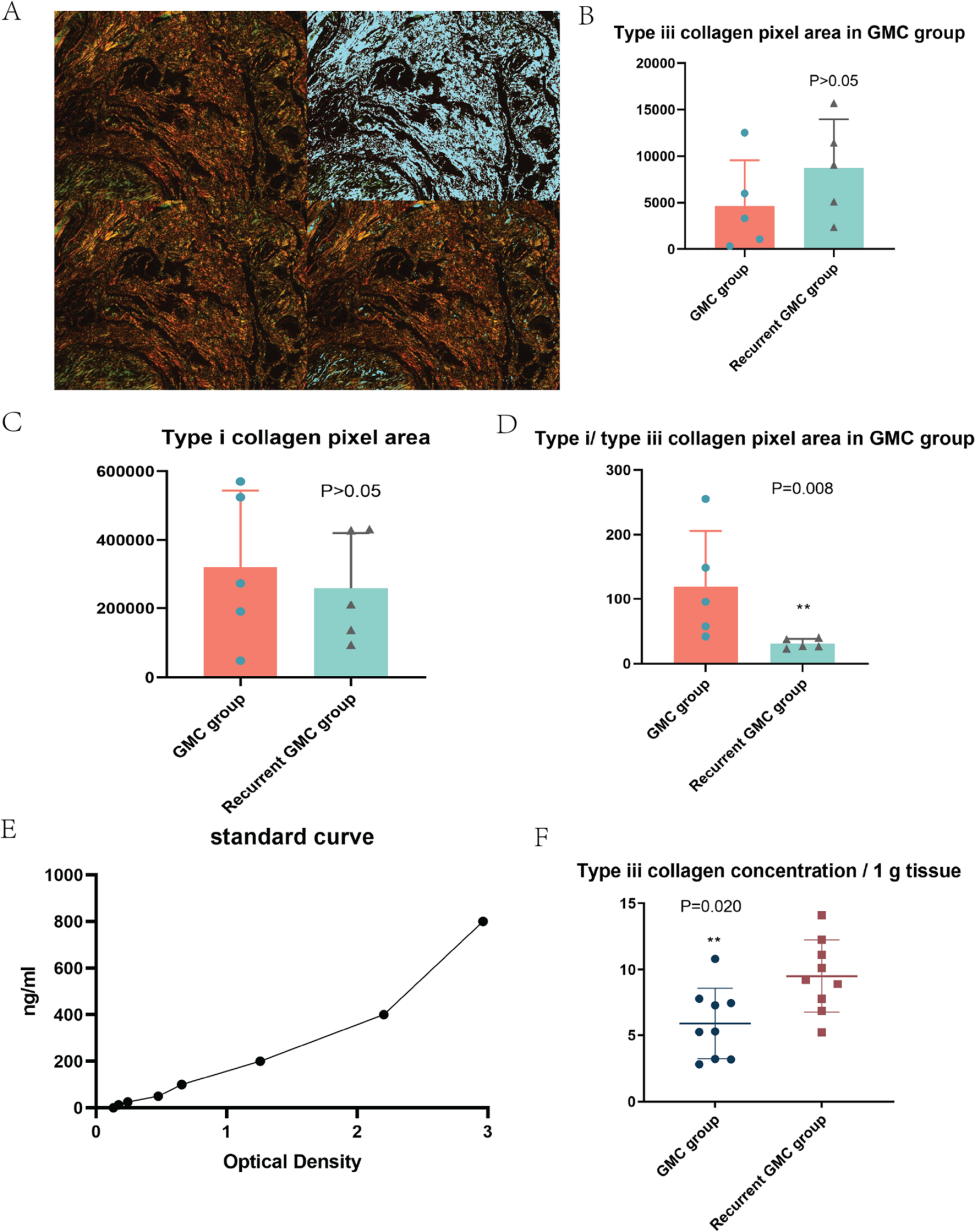
Contracture site had more type III collagen. **A** Area measurement method for type I and III collagen under polarized light for Sirius red-stained sections; three typical visual fields were selected from each slice for measurement, and the average value of the three visual fields was used for the final statistics. The unit is pixel. **B** Mann–Whitney *U* test results for type III and **C** type I collagen pixel area in GMC and recurrent GMC patients. **D** Mann–Whitney *U* test results for ratio of type I/III collagen in GMC and recurrent GMC patients. **E** Standard curve of the ELISA kit in use. **F** Mann–Whitney *U* test results for type III collagen concentration in 1 g contracture tissue in GMC and recurrent GMC patients

Figure 2A displays the typical calculation method of the area of type I and III collagen under polarized light. After imaging, we used Image-Pro Plus 6.0 software to measure the pixel area of type I collagen in three fields of view in each slice (1 on the right) with pixel area as the standard unit as well as the corresponding type III collagen pixel area (2 on the right). Then, we calculated the pixel area ratio of type I and type III collagen = type I collagen pixel area/type III collagen pixel area. The mean value of the three visual fields was used for the final statistics. There was no significant difference in the area of type I and type III collagen between recurrent GMC patients and GMC patients who underwent surgery for the first time (*P* > 0.05, Fig. 2B, C). Recurrent GMC patients seemed to have a lower type I/type III collagen ratio (*P* < 0.05, Fig. 2D). When comparing the content of type III collagen in the contracture tissue blocks, the recurrent GMC patients revealed elevated type III collagen concentration (*P* < 0.05, Fig. 2E).

### Collagen as a key factor for normal muscle repair in mouse gluteal muscle wound healing experiment

One reason for the formation of GMC is muscle damage and inflammatory stimulation caused by injection [12, 13, 23]. Tissue destruction and persistent inflammatory stimulation that exceed the body’s ability to repair will lead to sustained muscle defects and may result in tissue contracture. For this reason, we used the micro-deficient wound animal model to investigate the relationship between tissue repair errors and fibrosis. Specifically, we analyzed the single-cell RNA sequencing dataset (GSE GSE163376) through the GEO database19. This dataset reflects the administration of bilateral full-thickness 2 mm and 3 mm punch biopsy defects to the rectus femoris of adult C57BL6/J mice to model regenerative and degenerative muscle healing outcomes. According to the research findings, the 3 mm muscle defects failed to resolve until 42 days after surgery (subsequently termed degenerative defects), whereas regenerating myofibers filled the 2 mm defects 28 days after surgery. Compared to regenerative defects, degenerative defects were further characterized by higher proportions of smaller fibers. Mouse quadriceps in the injured site were extracted, and a single-cell solution was made using the quadriceps. An equal number of viable cells were pooled from two mice, and 10,000–16,000 cells were loaded into the 10 × Genomics Chromium Single Cell Controller; single cells were captured into nanoliter-scale gel bead-in-emulsions (GEMs). In addition, cDNAs were prepared using the Single Cell 3′ Protocol as per the manufacturer’s instructions and sequenced on a Nova Seq 6000 (Illumina) with 26 bases for read1 and 98 × 8 bases for read2. Except for 7-day, which was repeated twice by pooling two mice of the same gender, sequencing libraries were generated from a pool of cells from one male and one female mouse.

**Fig. 3:**
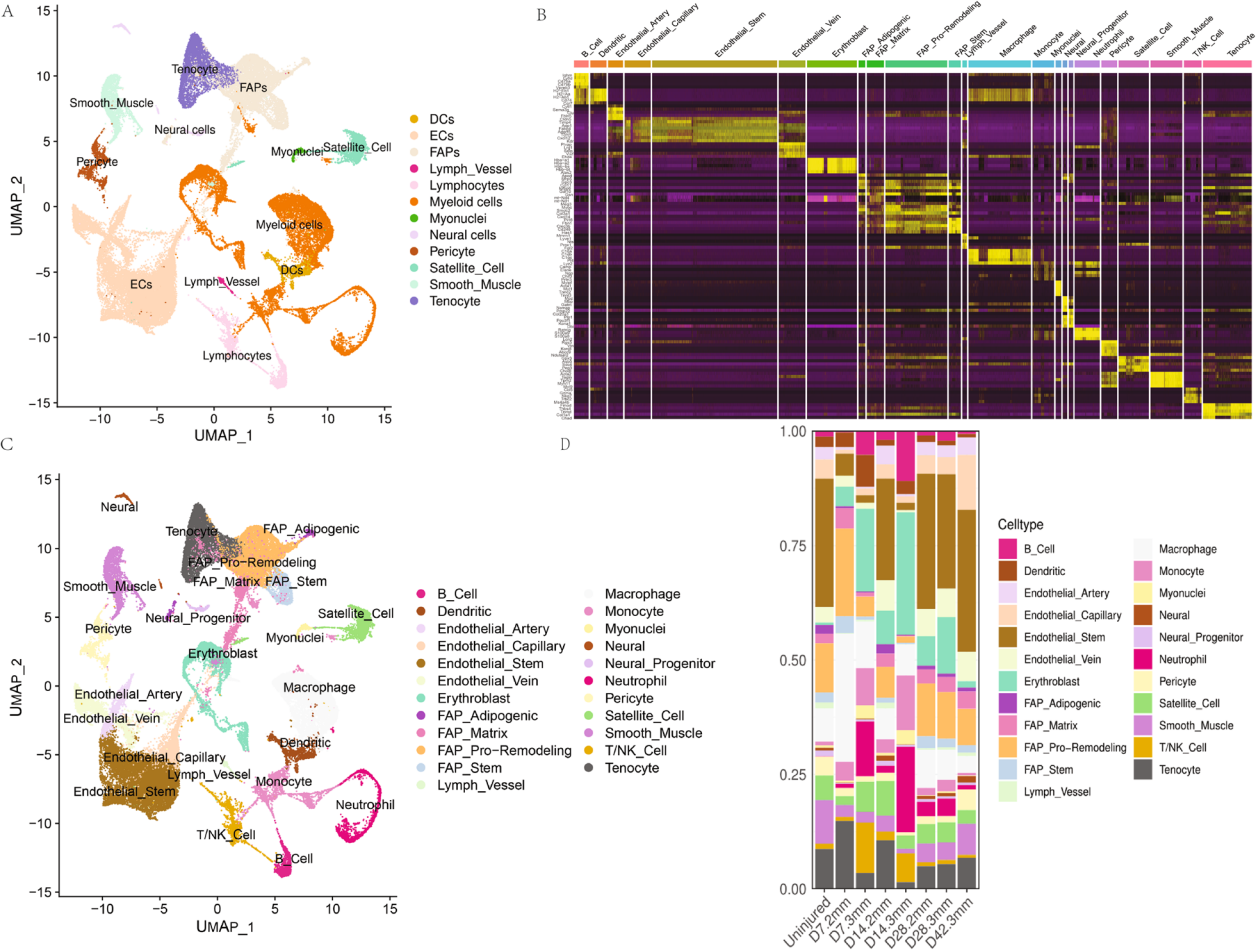
Single-cell RNA-seq results of mouse gluteal muscle wound healing model. **A** The Umap shows dimensionality reduction clustering and annotation of all cells. **B** The heatmap shows the cell marker gene expression in each cluster. **C** The Umap shows dimensionality reduction clustering and annotation of all sub-type cells. **D** Proportions of cell subsets in all samples. “2 mm” represents that the wound size created at the beginning was 2 mm. “3 mm” denotes that the size of the wound at the beginning of the experiment was 3 mm. The samples were taken 7, 14, 28, and 42 days after the wound was made. “Uninjured” indicates normal mouse gluteal muscle tissue without any surgery

The quadricep tissue was annotated into 12 major cell types, including dendritic cells (DCs), endothelial cells (ECs), fibroblastic adipogenic precursor cells (FAPs), lymph vessel cells, lymphocytes, myeloid cells, neural cells, pericytes, satellite cells, smooth muscle cells, and tenocytes, according to marker gene expressions (Fig. 3A, B). These major clusters were further annotated into 23 specific cell types (Fig. 3B, C). Figure 3D presents the cell proportions in each sample. Compared with uninjured muscle tissue, there were more immune cells in the early stage of injured tissue and more solid cells in the middle stage of injured tissue. At 28 days, the proportion of solid cells in the 2 mm wound was close to that of uninjured muscle tissue. The 3 mm wound tissue demonstrated a different growth pattern from that of the 2 mm wound tissue throughout the whole process, including fewer immune cells (macrophages, NK, and T cells) in the initial stage, more red blood cells, and fewer solid cells in the repair stage. At 42 days, the 2 mm wound was fully recovered, while the 3 mm wound was still in the defect state, which was also reflected in the observation that compared with uninjured tissue, day_ 42_3 mm tissue had more vascular tissue (ECs) and less muscle tissue (smooth muscle cells and tenocytes).

**Fig. 4:**
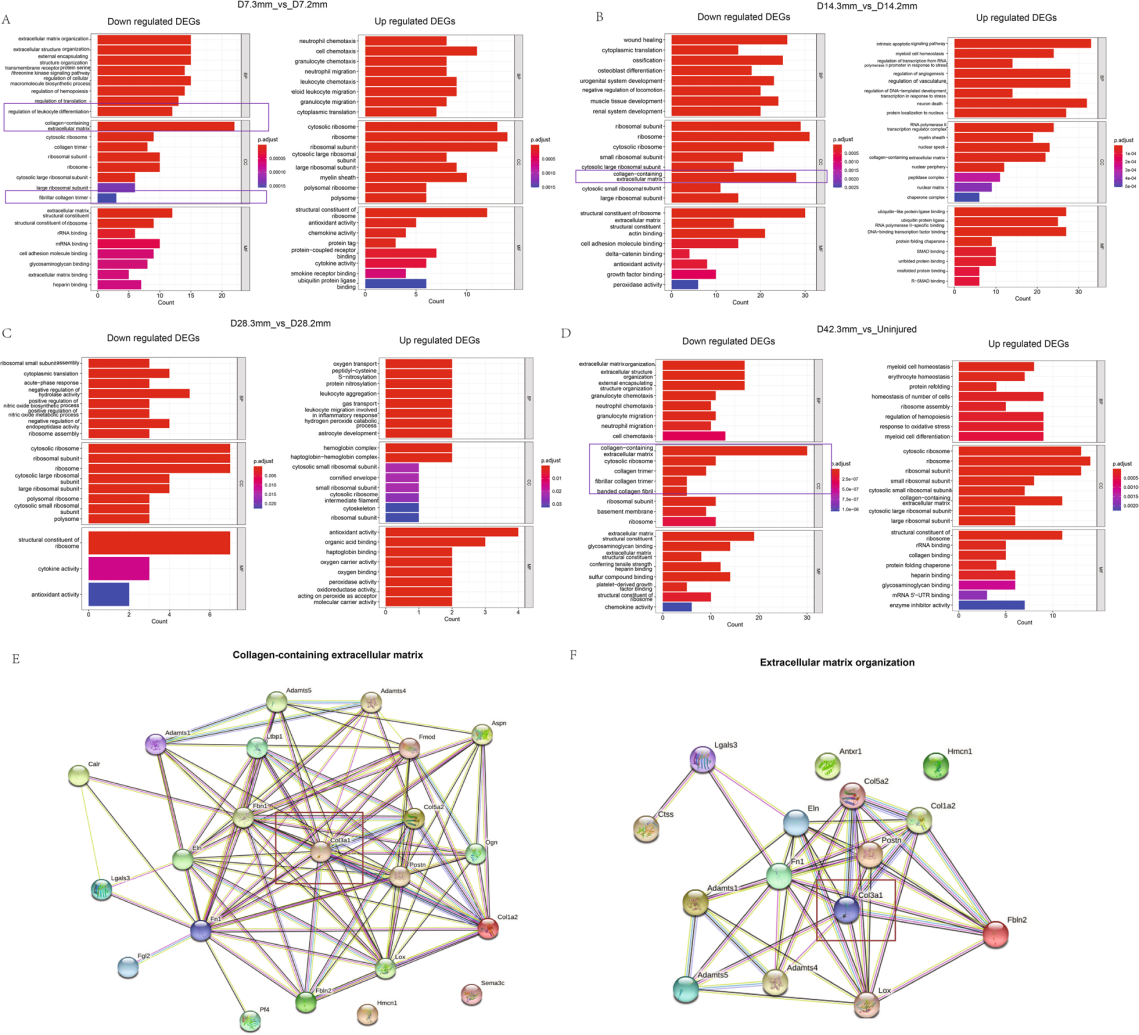
The way fibroblasts synthesize collagen may be the reason why muscle tissue cannot recover normally. **A** The GO items of DEGs between 3 mm scar muscle tissue and 2 mm scar muscle tissue on the 7th day after the wound was made. **B** The GO items of DEGs between 3 mm scar muscle tissue and 2 mm scar muscle tissue on the 14th day after the wound was made. **C** The GO items of DEGs between 3 mm scar muscle tissue and 2 mm scar muscle tissue on the 28th day after the wound was made. **D** The GO items of DEGs between 3 mm scar muscle tissue and uninjured muscle tissue on the 7th day after the wound was made. “2 mm” denotes that the wound size created at the beginning was 2 mm. “3 mm” indicates that the size of the wound at the beginning of the experiment was 3 mm. The samples were taken 7, 14, 28, and 42 days after the wound was made. “Uninjured” represents normal mouse gluteal muscle tissue without any surgery. Protein interaction results in two GO pathways E. collagen-containing extracellular matrix and **F** extracellular matrix organization

Interestingly, we did not see a significant difference in the proportion of FAPs between the 3 mm wound and 2 mm wound at 28 days, as well as the 3 mm wound and undamaged tissue at 42 days. We suspect that fibroblast lineage was through functional changes rather than simple quantitative changes that altered the outcome of quadriceps femoris wound recovery. Therefore, we compared the DEGs between different samples in all the FAPs. Specifically, we performed GO enrichment for the up- and down-regulated DEGs (Fig. 4A–D). Concerning the top 8 GO items in the cellular component category (CC), at the wound healing early stage (day 7 and day 14), the down-regulated genes in the 3 mm wound enriched mainly in collagen-related pathways, such as the collagen-containing extracellular matrix (Fig. 4E, F), collagen trimer, and fibrillar collagen trimer compared with 2 mm tissue. In addition, at the wound healing late stage, compared with uninjured tissue, the down-regulated genes in the 3 mm wound also enriched mainly in collagen-related pathways, such as the collagen-containing extracellular matrix, collagen trimer, fibrillar collagen trimer, and banded collagen fibril. This result is consistent with the pathological results we previously observed in the contracture tissue of GMC patients and the wound tissue of mice.

Whether a muscle injury can embark on the normal recovery process and eventually be repaired is closely related to the collagen secretion function of fibroblast lineage. Failure to produce normal muscle fiber collagen may be one reason why a wound cannot recover and eventually leads to a defect.

From the results of GO enrichment, the healing difference in gluteal muscle wounds was obvious in the early stage (day 7). Therefore, we further analyzed two important GO items in early wound healing, the gene interaction network for the collagen containing extracellular matrix (containing 22 items) and extracellular matrix organization (containing 15 items), using the STRING database. We found that the core gene that interacts most with other genes is COL3A1, which regulates type III collagen. This finding indicates that the type of collagen, especially the proportion of type III collagen, may be critical in the process of injury recovery. In addition to collagen, immune response (chemotaxis-related pathways), oxygen transport, extracellular matrix, and other pathways were observed to play a specific role in whether muscle wounds can recover normally.

## Discussion

Gluteal muscle contracture is a syndrome involving contracture and distortion of the gluteal muscles (mainly in the gluteus maximus) and fascia fibers [4]. The contracture of the gluteus maximus results in hip dysfunction on flexion, adduction, internal rotation, clinical signs of positive out-toe gait, cross-leg test, and squatting with side-by-side knee test [24]. Operative management includes open surgery and arthroscopic release [19]. Previous reports focused on the good practice of arthroscopic release and its advantages over open surgery [7–9]. The follow-up of arthroscopic release’s outcome is insufficient, and there is insufficient attention to recurrence, especially for patients with severe GMC. GMC patients are divided into three grades according to clinical symptoms and imaging results. Among these categories, GMC III was characterized by more severe symptoms, longer postoperative hospital stays, and worse postoperative physical examination results in our cohort [12, 25, 26]. In our cohort, compared with GMC II group, the GMC III group had more patients with unequal length of lower limbs. And GMC III group displayed worse JOA scores and restricted hip movement. At six weeks after the surgery, compared with GMC II patients, GMC III patients had a higher obvious hip snap rate, lower JOA score, and worse hip range of motion. These results suggest that the prognosis may be inversely proportional to the preoperative severity.

Regarding the physiological structure, the superficial fibers and the deep upper part of the gluteus maximus end in a tendinous sheet that passes lateral to the greater trochanter and is attached to the iliotibial band of the fascia. In contrast, the lower part of the muscle’s deep fibers is inserted into the greater trochanter. Muscle degeneration and fibrillation cause further gluteal muscle contracture, eventually leading to GMC syndrome. Consistent with the previous literature, we found that the compared contracture tissue lacked normal muscle morphology and fiber status. Instead of muscle collagen fibers, the contracture tissue tended to have more type III collagen and form shorter fibers. In a further comparison of the pathological difference of the contracture tissues between primary GMC and recurrent GMC patients, the recurrent GMC patients exhibited more type III collagen. Type III collagen is synthesized by cells as a pre-procollagen, which is also found as a major structural component in hollow organs, such as large blood vessels, the uterus, and the bowel, all of which are tissues that must withstand stretching [27]. This collagen type dominates the early phases of wound healing and granulation tissue formation. However, in the late muscle regeneration stage, too much type III collagen is unsuitable for wound recovery.

One previous study confirmed that collagen types I, III, TGF-b1, and TGF-b3 were up-regulated in biopsy specimens obtained from GMC patients in comparison with the unaffected adjacent muscle [14]. Recent studies have indicated that exosomes and exosomal miRNAs from muscle-derived fibroblasts also play a role in skeletal muscle fibro-sis [28]. Fibroblasts are developmentally programmed to produce a collagen matrix, which is crucial for tissue repair. Faulty tissue repair pathways are one cause of muscle fibrosis [15]. Several studies have supported the relationship between fibroblast and muscle fibrosis, including transgenic animal studies and fibrosis histopathological studies [29, 30]. Similar with other muscle fibrosis diseases, the occurrence of GMC is related to the tissue repair error after injection [12, 25]. Following acute muscle injury, infiltrating inflammatory cells and resident stem cells orchestrate their activities to restore tissue homeostasis. However, in the muscle repair failure, the inflammatory-cell infiltration and fibroblast activation persists, while the reparative capacity of stem cells (satellite cells) is attenuated [31]. Many evidences supported that the muscle fibrosis caused by trauma is the product of the late stage of inflammation, and different reports point out that the fate change of fibroblasts during tissue repair is very important for muscle fibrosis (abnormal repair) [32, 33].

Artificial muscle defect model is a good model of study on the process of muscle repair. In the artificial muscle defect model, compared with the repairable wound (2 mm), the irreparable wound (3 mm) showed down-regulated collagen-related pathways in the early stage. Compared with normal muscle, the irreparable wound also showed a defect in collagen-related pathways. At the same time, imbalanced immune regulation and oxygen might reflect the muscle defect repair. The only substantial evidence of a pathogenic cause of GMC to date has been a history of repeated intramuscular injection [8, 13]. Through bioinformatics analysis results and pathological experiments, we hypothesized that one of the possible causes of GMC might be that the muscle defect exceeds its repairability. In addition, chronic inflammation may trigger pathological changes, making repeated intramuscular injection a risk factor.

In general, this study compared the differences in preoperative and postoperative indicators among GMC patients who undergo arthroscopic release with different severity in detail for the first time. And this study found that the fibrous and collagen composition of contracture sites in GMC patients was very different from that of normal muscle. Patients with relapsed GMC had more type III collagen and shorter collagen fibers. The results of single-cell RNA sequencing analysis suggested that the collagen secretion function of fibroblasts may be closely related to the typical recovery of muscles. One limitation of this study was the difficulty in obtaining contracture tissue, resulting in a small number of samples. Another limitation was the inability to obtain the normal muscle of the patient as a control.

### Supplementary Information

**Additional file 1**. High-definition Images and Ethical.

## Data Availability

The data underlying this article are available in the article and in its Additional file 1.
