# Supplementary material for: The pattern of collagen production may contribute to the gluteal muscle contracture pathogenic process
Source: J Orthop Surg Res. 2023 Aug 8;18:579. doi: 10.1186/s13018-023-04069-w (PMC10408206; doi:10.1186/s13018-023-04069-w)
Supplement: Supplementary file 1 — Additional file 1. High-definition Images and Ethical. [file 13018_2023_4069_MOESM1_ESM.zip › ╨┬╜¿╬─╝■╝╨/No.XJS2017008.pdf]

北京大学深圳医院伦理审查批件

批件号：北大深医伦审（研）[2017]第(008)号

项目名称	臀肌挛缩症规范化诊断、治疗与康复的临床研究		
项目来源	课题申报		
项目负责人	张新涛	所在科室	康复医学科
申办方/资助方	北京大学深圳医院（资助）	CRO	不适用
审查文件	研究方案（v1，2017-10-14）<br>知情同意书（v1，2017-10-14）		
批准文件	研究方案（v1，2017-10-14）<br>知情同意书（v1，2017-10-14）		
审查类别	☑初始审查	□跟踪审查	□复审
审查方式	☑会议审查	□快速审查	
会议日期	2017-10-26	审查会议地点	综合楼四楼会议室（3）
投票结果	共有委员 21 名，实到 12 名，投票 12 名，回避 0 名		
	同意 12 票	作必要的修正后同意 0 票	作必要的修正后重审 0 票
	不同意 0 票	终止或暂停已批准的试验 0 票	
审查意见	同意		
	根据卫生部《涉及人的生物医学研究伦理审查办法》（2016 年）、CFDA《药物临床试验质量管理规范》（2003 年）、CFDA《药物临床试验伦理审查工作指导原则》（2010 年）、WMA《赫尔辛基宣言》和 CIOMS《人体生物医学研究国际道德指南》的伦理原则。经本伦理委员会审查，同意按所批准的临床研究方案、知情同意书开展本项研究。<br>注：<br>1、请遵循 GCP 原则、遵循伦理委员会批准的方案开展临床研究，保护受试者的健康和权利。<br>2、对研究方案、知情同意书、招募材料等的任何修改，请提交修正案审查申请。<br>3、发生 SAE，请及时提交严重不良事件报告。<br>4、如有不依从/违背方案的情况，请及时提交违背方案报告。<br>5、请根据年度/定期跟踪审查频率，及时提交研究进展报告。<br>6、暂停或提前终止临床研究，请及时提交暂停/终止研究报告。<br>7、完成临床研究，请提交结题报告。		
年度定期/跟踪审查频率	12 个月	批件有效期	三年<br>（请在批件过期前一个月提交跟踪审查申请）
联系人	曾杏珍	联系电话	0755-83923333-8809
主任委员（被授权者）签名			伦理委员会（盖章）<br>2017 年 10 月 26 日
