# Supplementary figures and images for: The pattern of collagen production may contribute to the gluteal muscle contracture pathogenic process
Source: J Orthop Surg Res. 2023 Aug 8;18:579. doi: 10.1186/s13018-023-04069-w (PMC10408206; doi:10.1186/s13018-023-04069-w)

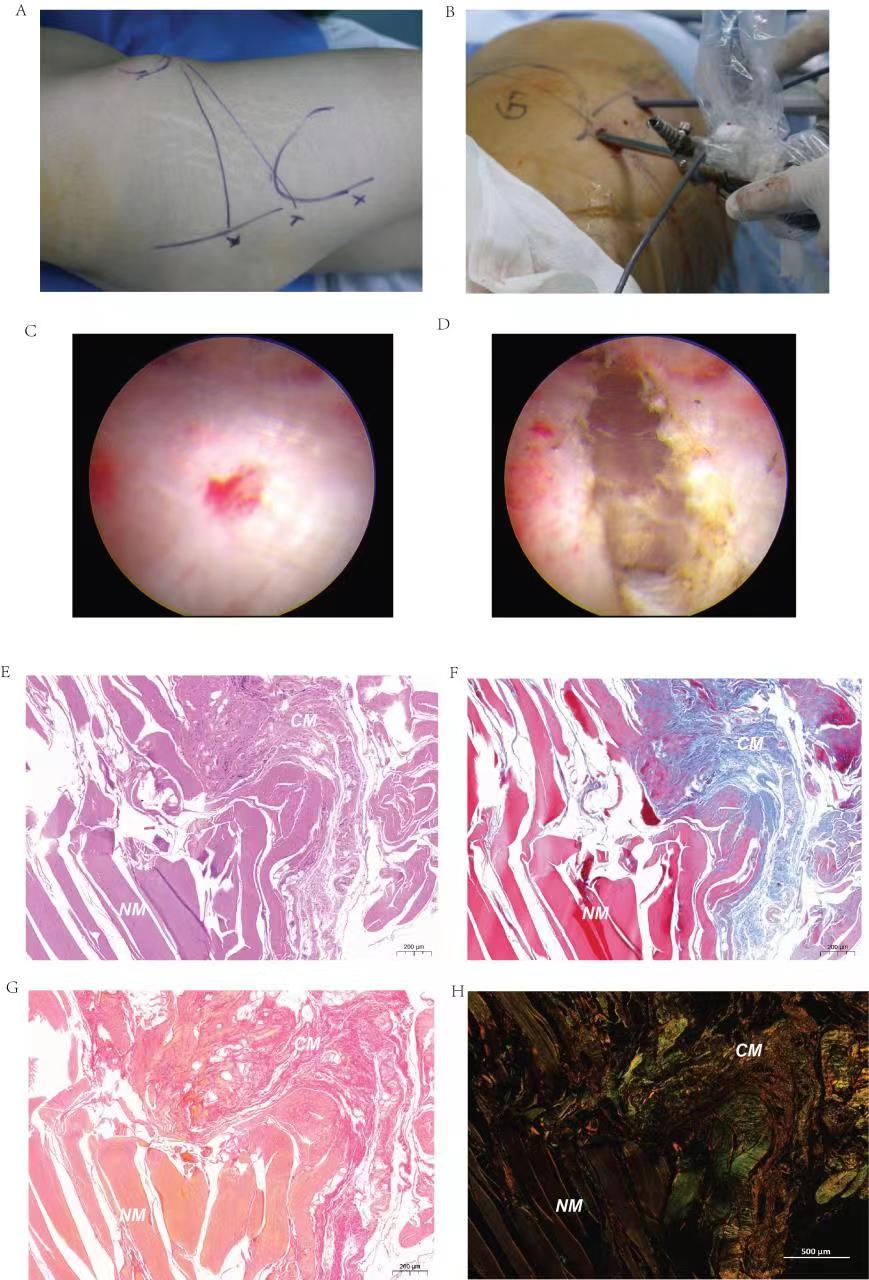

Supplement: Supplementary file 1 — Additional file 1. High-definition Images and Ethical. [file 13018_2023_4069_MOESM1_ESM.zip › ╨┬╜¿╬─╝■╝╨/figure 1.jpg]

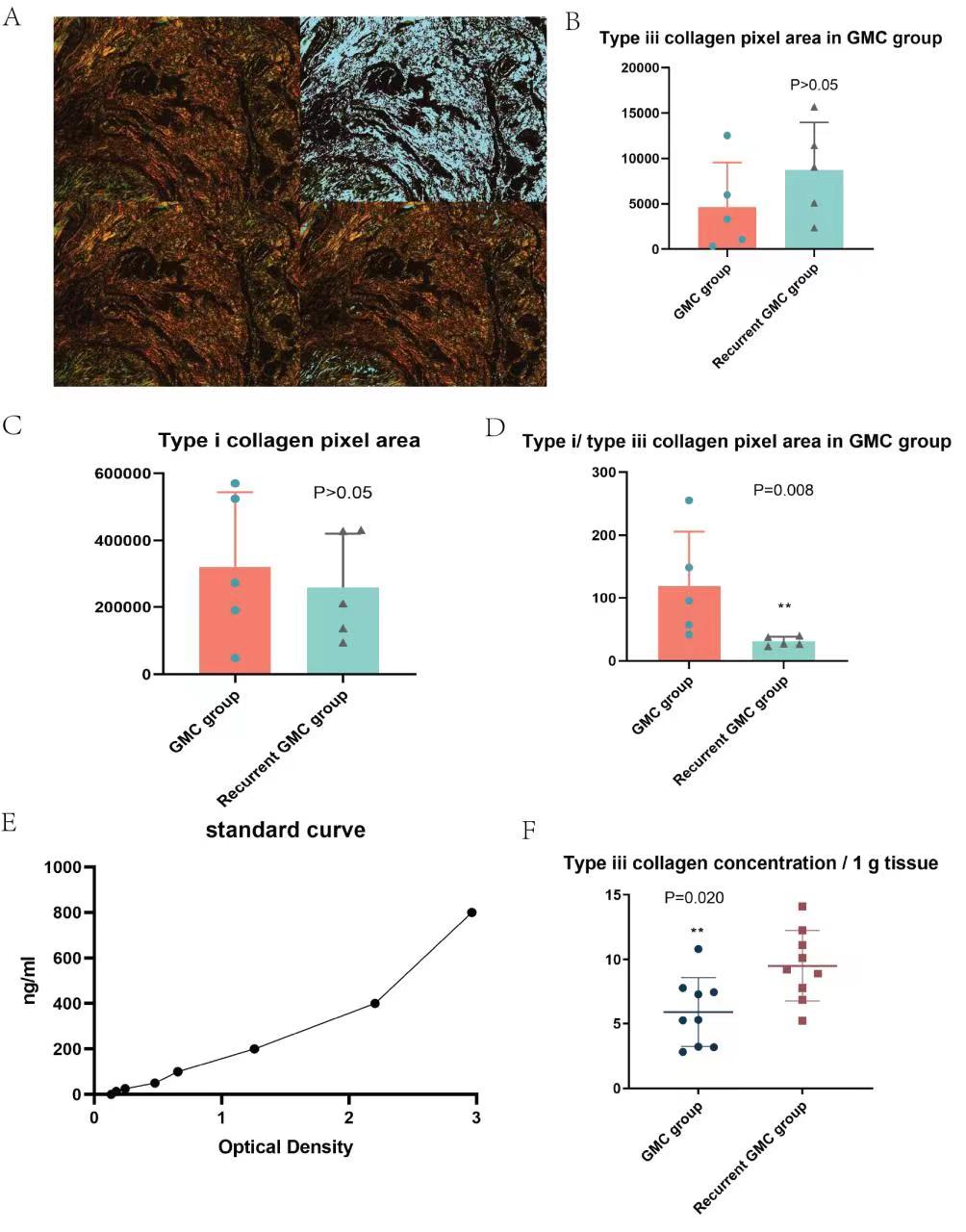

Supplement: Supplementary file 1 — Additional file 1. High-definition Images and Ethical. [file 13018_2023_4069_MOESM1_ESM.zip › ╨┬╜¿╬─╝■╝╨/figure 2.jpg]

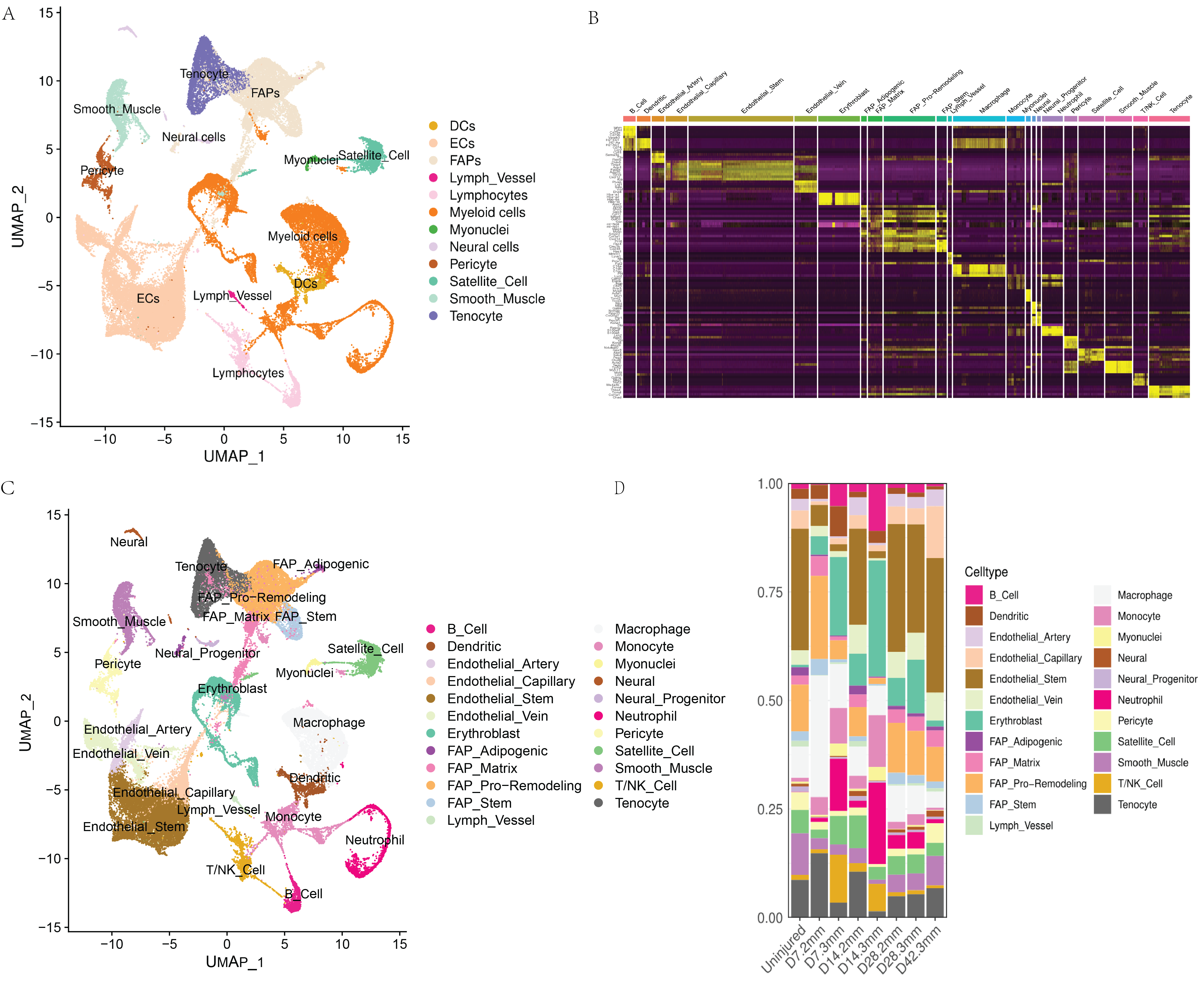

Supplement: Supplementary file 1 — Additional file 1. High-definition Images and Ethical. [file 13018_2023_4069_MOESM1_ESM.zip › ╨┬╜¿╬─╝■╝╨/Figure 3.png]

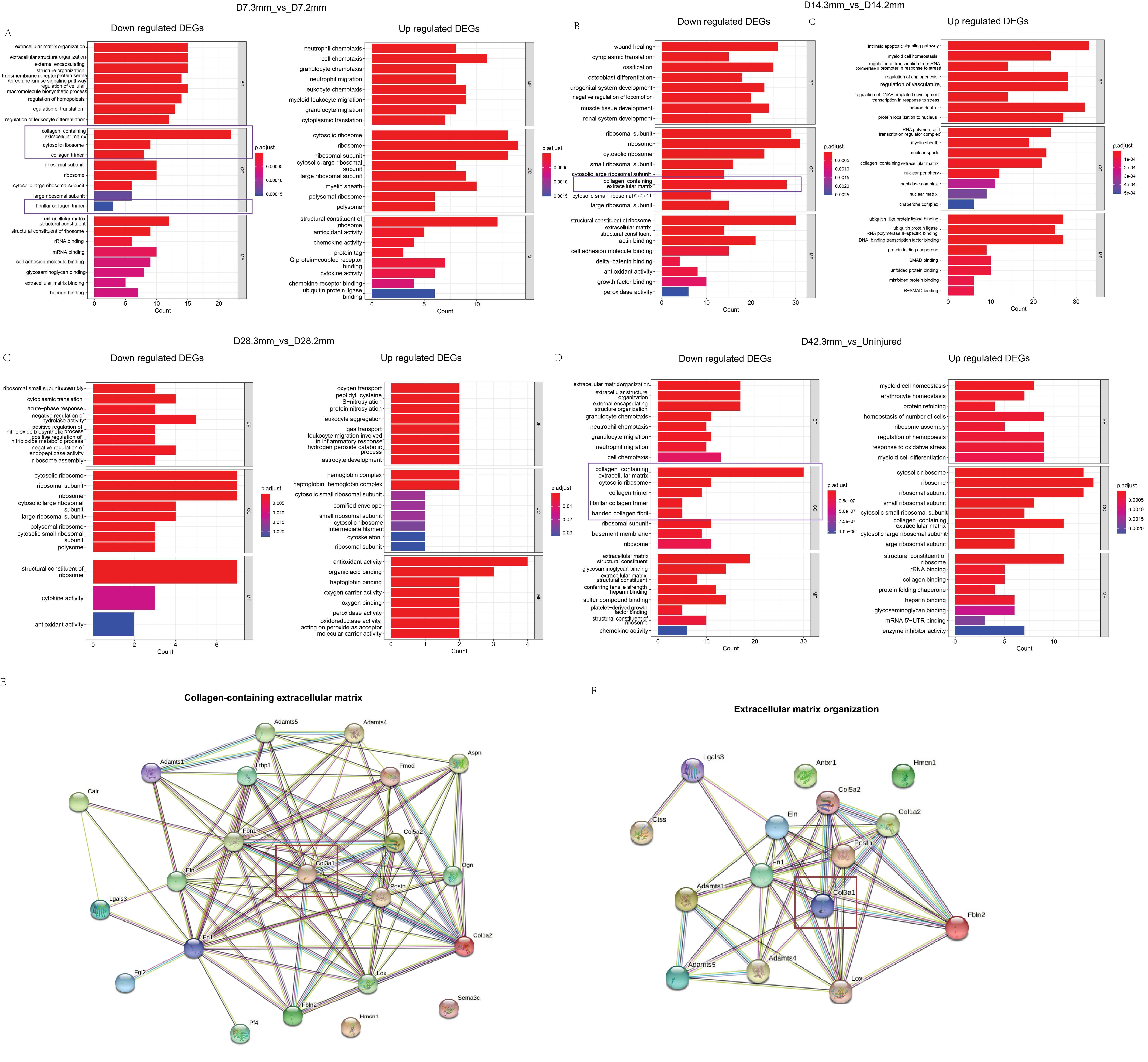

Supplement: Supplementary file 1 — Additional file 1. High-definition Images and Ethical. [file 13018_2023_4069_MOESM1_ESM.zip › ╨┬╜¿╬─╝■╝╨/Figure 4.png]
